# Effects of left atrial function on pulmonary arterial pressure in acute myocardial infarction, hypertrophic and dilated cardiomyopathy

**DOI:** 10.1186/s12872-022-02952-8

**Published:** 2022-11-26

**Authors:** Minjeong Kim, Hyemoon Chung, In-Soo Kim, Chul Hwan Park, Se-Joong Rim, Eui-Young Choi

**Affiliations:** 1 Division of Cardiology, Myongji Hospital, Hanyang University College of Medicine, Seoul, South Korea; 2 Division of Cardiology, Department of Internal Medicine, Kyung Hee University School of Medicine, Seoul, South Korea; 3 Division of Cardiology, Heart Center, Gangnam Severance Hospital, Yonsei University College of Medicine, 211 Eonju-Ro, Gangnam-Gu, Seoul, 06273 Republic of Korea; 4 Department of Radiology, Gangnam Severance Hospital, Yonsei University College of Medicine, Seoul, South Korea

**Keywords:** Left atrial function, Pulmonary artery systolic pressure, Dilated cardiomyopathy, Hypertrophic cardiomyopathy, Acute myocardial infarction

## Abstract

**Background:**

To investigate the differential contribution of the left atrial (LA) function and left ventricular (LV) fibrosis to pulmonary arterial systolic pressure (PASP) in hypertrophic cardiomyopathy (HCM), dilated cardiomyopathy (DCM) and reperfused acute myocardial infarction (AMI).

**Methods:**

Data of 370 patients with HCM (n = 133), DCM (n = 114) and reperfused AMI (n = 123) who underwent both echocardiography and cardiovascular magnetic resonance (CMR) were comprehensively reviewed. Phasic LA volumes, LA-global longitudinal strain (GLS), LA stiffness index, defined as E/e′/LA-GLS and extracellular volume fraction (ECV) of LV were measured using CMR.

**Results:**

E/e′ was correlated with PASP in all groups; however, the predicted value was significantly attenuated after adjusting for LA volume and LA strain in HCM and DCM, but remained significant in AMI. The LA stiffness index was related to PASP in HCM (*p* = 0.01) and DCM (*p* = 0.03) independent of LA volume index and E/e′, but not in AMI. In DCM, ECV was significantly related to PASP (*p* < 0.001) independent of LA volume index and E/e′. When subdivided according to the linear regression between PASP and E/e′, patients in the discrepantly high PASP group had lower total emptying fraction and reservoir fraction of left atrium in HCM and DCM but not in AMI.

**Conclusions:**

The LA function in HCM and DCM and LV fibrosis in DCM correlated with PASP independent of E/e′ and LA size, contrary to that in AMI. These results suggest the presence of LA dysfunction in non-ischemic cardiomyopathies and usefulness of ECV measurement in DCM for the comprehensive evaluation of LV diastolic function.

**Supplementary Information:**

The online version contains supplementary material available at 10.1186/s12872-022-02952-8.

## Background

Pulmonary pressure elevation is commonly observed in patients with left ventricular (LV) diastolic dysfunction. This is due to passive backward pressure transmission to the pulmonic vein through the left atrium due to an increased LV filling pressure. Currently, the echocardiographic methods for defining LV diastolic dysfunction are based on an early diastolic mitral annular velocity (e′), ratio of an early mitral inflow to e′ (E/e′), maximal left atrial (LA) volume index (LAVI), and an estimated pulmonary arterial systolic pressure (PASP) [1]. Ultimately, it focuses on the evaluation of elevated LV filling pressure, as the reason behind the measurement of diastolic function is to guide the preload reduction therapy in patients with elevated pulmonary capillary wedge pressure (PCWP) or to evaluate the degree of disease progression in the LV myocardium, such as interstitial fibrosis. However, among the four parameters, PASP by tricuspid regurgitant velocity (TRV) is the final result of LV diastolic dysfunction and represents PCWP unless a high flow status or combined pulmonary vascular disease is present. E/e′ is a well-known index that predicts PCWP elevation. However, recent studies have shown that its predictability is modest in advanced heart failure (HF) with reduced ejection fraction (HFrEF), including DCM and left bundle branch block, and hypertrophic cardiomyopathy (HCM) [2, 3]. In these patients, other non-invasive LV diastolic functional indices for predicting PCWP are needed.

The LA strain was found to correlate with invasively determined LV end diastolic pressure as well as the levels of N-terminal pro-B-type natriuretic peptide [4]. The LA volume index, used as a diastolic functional parameter, is the maximal volume index measured at the LV end-systole. This represents longstanding pressure or volume overload in the LA. However, the maximal LA volume index has been shown to be different in men and women and larger in athletes. Therefore, a minimal LA volume index or phasic LA function is more closely related to the LV diastolic function. The differential role of the LA reservoir, conduit, and booster pump functional indices for pulmonary pressure elevation in various myocardial diseases have not been thoroughly evaluated. Contrary to the indirect assessment by transmitral inflow indexes or LA functional parameters, direct LV tissue characterization provides true load-independent LV diastolic function. Cardiovascular magnetic resonance (CMR) was known to determine LA function such as LA fractional change or ejection fraction more exactly than conventional TTE. TTE also underestimates LA volume compared to CMR [5]. Furthermore, measurement of LA volume by CMR showed better reproducibility compared to TTE in patients with AF [6]. In estimating the LA function, there is another method by measuring LA strain in addition to the volumetric method. LA stiffness is known to be related to LA reservoir function and LV filling pressure, and increases with LA remodeling. The LA stiffness index was calculated as the ratio of E/e′ to LA global strain [7]. It was known to be superior to volume parameters in predicting diastolic dysfuction in HFpEF [7, 8]. The development of a T1 mapping technique in CMR could provide an accurate extracellular volume fraction (ECV) representing the degree of diffuse interstitial fibrosis [9], which is closely related to impaired active relaxation and increased passive stiffness of the left ventricle [10, 11]. Therefore, in this study, we sought to evaluate the role of LA function measured by CMR for the development of PASP in HCM (prototype of HFpEF), DCM (prototype of HFrEF), and reperfused AMI (prototype without longstanding diastolic dysfunction) patients. In addition, using the ECV of CMR, the relationship between the LV myocardial structural changes and PASP was evaluated.

## Methods

### Study population

We reviewed the clinical, TTE, and CMR findings of 370 patients at a single tertiary center, who were diagnosed with reperfused AMI, HCM, or DCM. The inclusion criteria were as follows: (1) patients with a first AMI who underwent successful percutaneous coronary intervention within 48 h of chest pain were enrolled, AMI was diagnosed on the basis of the elevated levels of cardiac enzyme and ST-segment or T-wave deviation on electrocardiography according to the established diagnostic criteria [12]. We excluded patients with chronic ischemic disease. Average duration after onset of AMI to CMR was 2.4 ± 2.6 days after revascularization. (2) Regarding culprit vessels, 82 (66.7%) had in left anterior descending coronary artery (LAD), 8 (6.5%) in left circumflex coronary artery and 33 (26.8%) in right coronary artery territories (Additional file 1: table S1). Patients with maximal LV hypertrophy greater than 13 mm and a ratio of maximal thickness to posterior wall thickness greater than 1.3 without an underlying cause of hypertrophy, such as uncontrolled hypertension or aortic stenosis, were enrolled as HCM [13]. The patients with a diagnosis of infiltrative disease, such as amyloidosis were excluded in HCM group. (3) DCM patients were defined individuals who matched the diagnostic criteria established by the World Health Organization. The patients diagnosed with non-ischemic cause of DCM were only included. The patients with inadequate tracking quality by CMR and those with pulmonary vascular disease at the time of echocardiography or CMR were also excluded. This study was approved by the Institutional Review Board of Gangnam Severance Hospital (3-2021-0030).

### Echocardiographic analysis

A comprehensive echo-Doppler evaluation was performed according to the current guidelines [14]. The E/e′ ratio was subsequently calculated. When the peaks E and A were fused due to tachycardia or atrioventricular block, the peak velocities of the fused mitral inflow waves were used for E/e′ calculation [15]; additionally, the average value of five consecutive beats was used in patients with atrial fibrillation. The PASP was calculated as 4 × (peak TRV)^2^ + right atrial pressure, where the right atrial pressure was estimated according to the inferior vena cava diameter and its respiratory variations.

### CMR imaging and measurements of ECV

**Fig. 1:**
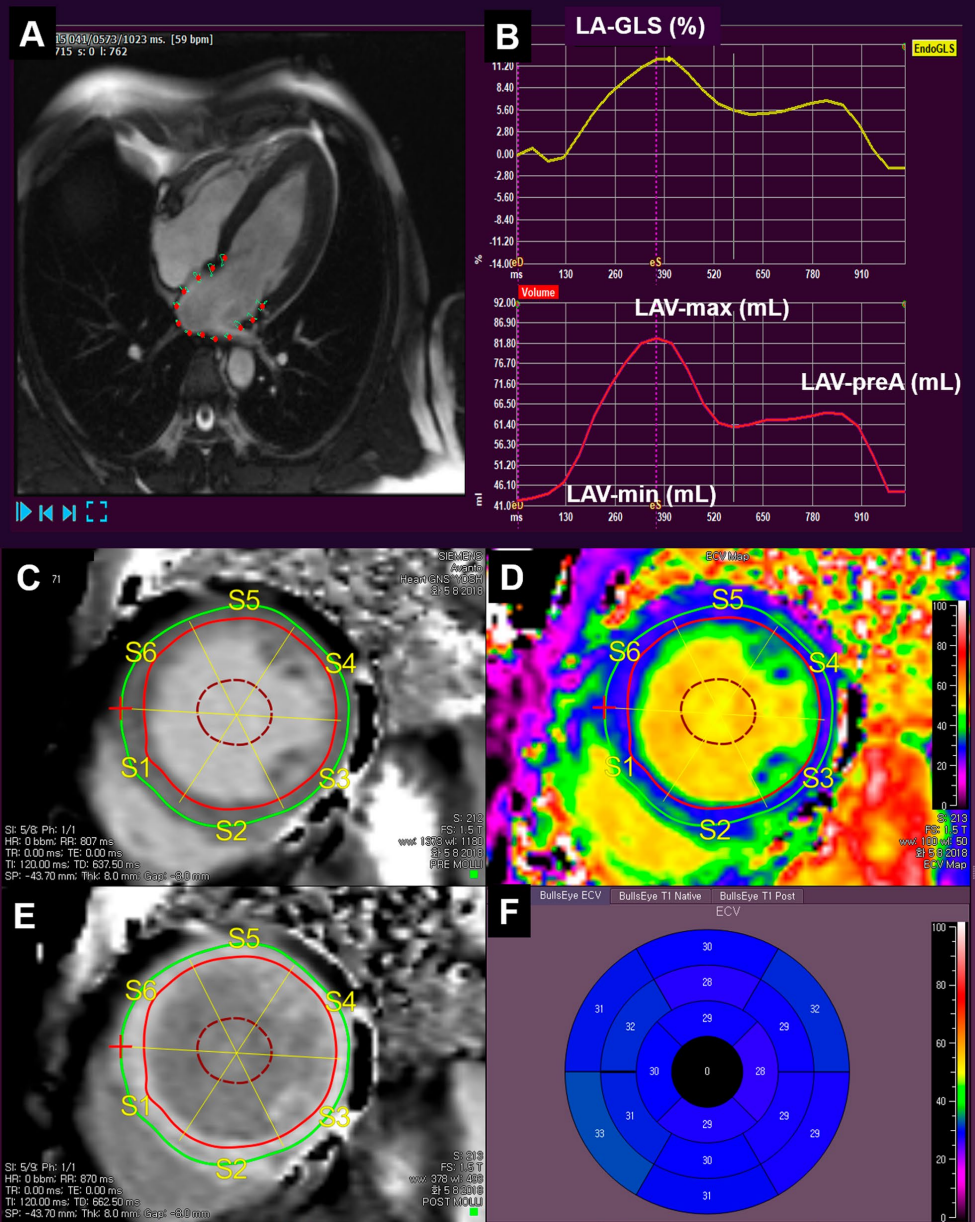
The feature tracking analysis of the left atrium and assessment of left ventricular extracellular volume fraction (ECV). Tracking of left atrial motion (**A**), global longitudinal strain (**B**, upper panel), and phasic volumes (**B**, lower panel). Endocardial, epicardial borders and cavity tracings and segmentation in native T1 map (**C**) and post-T1 map (**E**). The reconstructed color coded ECV map (**D**) and its 16-segmental values in the bull′s eye map (**F**). LA, left atrial; GLS, global longitudinal strain; LAV-max, maximal LA volume; LAV-min, minimal LA volume; LAV-preA, LA volume at the pre-atrial contraction phase

CMR was performed using a 1.5-T scanner (Magnetom Avanto; Siemens Medical Solutions, Erlangen, Germany) or 3-T scanner (Magnetom Vida; Siemens Medical Solutions, Erlangen, Germany) with a phased-array body coil. The LV 2-, 3-, 4-chamber, and short-axis views were obtained using cine images with a steady-state free precession sequence. After the administration of a gadolinium-based contrast agent (0.2 mmol/kg gadoterate dimeglumine; Dotarem, Guerbet, France), Native T1 mapping with a modified Look-Locker technique was performed during the mid-diastolic phase, and post-T1 mapping was performed 15 min after the contrast media injection using the same slice axis and parameters as the pre-T1 mapping. Native T1, post-T1, and ECV analyses were performed with QmapECV 2.2.44 (Medis, Leiden, Netherlands) [16]. The myocardial ECV (n = 297) was automatically calculated using the following equation in 16 segments, and the average value was used for further analysis.

$${\text{ECV}} = (\Delta {\text{R}}1\,{\text{of}}\,{\text{myocardium}}/\Delta {\text{R}}1\,{\text{of}}\,{\text{LV}}\,{\text{blood}}\,{\text{pool}}) \times \left( {1 - {\text{hematocrit}}} \right),$$

where R1=1/T1 and ${\Delta }$R1=post-contrast R1–pre-contrast R1 (Fig. 1).

The reproducibility and standardization of ECV measurement was reported previously [16].

### LA strain and phasic volume analysis using feature tracking CMR

The myocardial strain analysis using feature tracking CMR was performed using semi-automated software (QstrainMR 2.0, Medis, Leiden, Netherlands). The LA endocardial border was manually traced in a 4-chamber long-axis view using the LV end-diastole as the reference phase. The LA global longitudinal strain (GLS) was defined as the average peak strain value. The LA maximal, pre-contraction (pre-A in cases without atrial fibrillation), and minimal volumes were measured. The LA total emptying fraction was calculated as (LA maximal volume − LA minimal volume)/LA maximal volume; the reservoir fraction, as (LA maximal volume − LA minimal volume/LA minimal volume); the conduit fraction as (LA maximal volume − LA pre-A volume)/LA maximal volume; and the active emptying fraction as (LA pre-A volume − LA minimal volume)/LA pre-A volume [17], as shown in Fig. 1. The LA volume divided by the body surface area was defined as the LA volume index (LAVI), and E/e′ divided by LA-GLS was defined as the LA stiffness index [7].

### Statistical analysis

The baseline characteristics were summarized using frequencies and percentages and examined using the chi-square test for categorical variables. The continuous variables are reported as the mean and standard deviation or interquartile range for non-normally distributed variables. For normally distributed variables, the analysis of variance (ANOVA) was performed to test the differences among the three groups. A post-hoc test with Tukey’s HSD was conducted for pairwise comparisons. Non-normally distributed variables were compared using the Kruskal–Wallis test, and the Dunn’s post-hoc test was performed for pair-wise comparisons. A univariable linear regression model was used to estimate the unadjusted coefficient of primary endpoints for each echocardiographic and each CMR characteristic. Univariable factors with *p* < 0.05 and the major relevant clinical factors were entered into multivariable analyses for the predictive value of variables for PASP. The coefficient values were generated and expressed, together with their 95% confidence intervals. A subgroup analysis was performed to evaluate the differential effect of clinical variables among the three groups clustered by a linear regression between PASP and E/e′: (1) relatively higher PASP group compared to E/e′, (2) within 95% confidence interval and (3) relatively lower PASP group compared to E/e′. All analyses used two-tailed tests with a significance level of 0.05. Statistical analyses were performed using SPSS software (Statistical Package for Social Sciences, version 25.0, IBM Corp., Armonk, NY).

## Results

### Comparisons of the baseline characteristics and LA function between three groups

Among 370 patients who underwent both echocardiography and CMR, 133 (36%) patients had HCM, 123 (33%) patients had a history of reperfused AMI, and 114 (31%) patients had DCM. A total of 294 (80%) patients were men, and the average age was 56 ± 14 years. A total of 42 (11%) patients had atrial fibrillation. Patients with DCM were significantly younger than those with HCM (*p* < 0.05), and the prevalence of atrial fibrillation was significantly higher in DCM patients than in HCM and AMI patients. However, there were no significant differences in sex, systolic blood pressure, history of diabetes, and hypertension among the three groups. In the DCM group, a′ was significantly lower than that in the HCM and AMI groups. The LV ejection fraction and s′ were also significantly lower in the DCM group than in the other two groups. The PASP was significantly higher in the DCM group than in the other two groups, and the HCM group had a higher PASP than the AMI group. Likewise, the E/e′ of the DCM and HCM groups was significantly higher than that of the AMI group. The e′ of DCM and HCM was also significantly lower than that of the AMI group.

The values of LAVImax and LAVIpreA were significantly higher in the DCM and HCM groups than in the AMI group. The GLS of the LA was significantly reduced, and the LA stiffness index was significantly higher in the DCM group than in the other groups. The LA total emptying fraction, LA active emptying fraction, reservoir fraction, and conduit fraction were serially decreased in the AMI, HCM, and DCM groups. The average ECV appeared to be lower in the DCM and HCM groups than in the AMI group (Table 1). In HCM and AMI, LV-ECV was most significantly correlated with LA stiffness index but in DCM, LV-ECV was most significantly correlated with LA maximal volume index (Additional file 1: table S2).The LA-pre-A volume index could be calculated only in 310 patients after the exclusion of atrial fibrillation (n = 42) and non-measurable pre-A volume in phasic LA volume curves (n = 18, 13 in DCM; 4 in AMI; and 1 in HCM).

**Table 1:** The baseline clinical, echocardiographic, and cardiovascular magnetic resonance findings according to the disease

Variables	HCM (n = 133)	AMI (n = 123)	DCM (n = 114)	*p* value
Age (years)	58.5 ± 13.1	55.7 ± 12.2	54.3 ± 15.3*	0.04
Male [n (%)]	97 (73)	111 (90)*^†^	86 (75)	0.001
Body surface area (m^2^)	1.8 ± 0.2	1.8 ± 0.2	1.8 ± 0.3	0.17
Systolic BP (mmHg)	118 (107, 135)	114 (103, 129)	118 (103, 132)	0.10
Diastolic BP (mmHg)	72 (62, 80)	71 (65, 79)^†^	78 (67, 89)*	0.001
NYHA class	1.42 ± 0.59	1.62 ± 0.61*^†^	2.42 ± 0.65*	< 0.001
Diabetes [n (%)]	25 (19)	27 (22)	20 (18)	0.67
Hypertension [n (%)]	71 (53)	58 (47)	48 (42)	0.21
ACEi/ARB [n (%)]	69 (52)	101 (82)*	96 (84)*	< 0.001
Beta-blocker [n (%)]	92 (69)	104 (85)*	93 (82)	0.01
Diuretics [n (%)]	28 (21)	10 (8)*^†^	81 (71)*	< 0.001
^1^Atrial fibrillation [n (%)]	14 (11)	3 (2)*^†^	25 (22)*	< 0.001
^2^NT-proBNP (pg/mL)	711.9 ± 1111.9		4845.1 ± 7338..4*	0.004
^2^BNP (pg/mL)		141.3 ± 255.2^†^	1389.5 ± 1233.3	< 0.001
eGFR (mL/m^2^/1.73 m^2^)	82.4 ± 13.5	93.0 ± 23.6*^†^	83.5 ± 18.3	< 0.001
Echocardiography				
Relative wall thickness	0.48 (0.42, 0.55)	0.42 (0.38, 0.48)*^†^	0.31 (0.27, 0.36)*	< 0.001
e′ (cm/s)	5 (4, 6)	6 (5, 7)*^†^	5 (4, 7)	< 0.001
a′ (cm/s)	8 (7, 9)	9 (7, 10)*^†^	5 (3, 7)*	< 0.001
s′ (cm/s)	7 (6, 8)	7 (6, 8)^†^	4 (3, 5)*	< 0.001
E/e′	13.6 (10.6, 18.5)	10.6 (8.8, 13.4)*^†^	16.0 (11.9, 22.3)	< 0.001
PASP (mmHg)	26.7 (23.4, 31.0)	23.8 (19.4, 28.1)*^†^	38.1 (24.2, 46.8)*	< 0.001
LAVI (mL/m^2^)	33.7 (27.7, 46.4)	21.7 (17.4, 26.6)*^†^	49.5 (36.2, 63.9)*	< 0.001
LVMI (g/m^2^)	119.8 (101.6, 143.0)	93.14 (77.9, 106.2)*^†^	129.0 (104.8, 149.1)	< 0.001
LV ejection fraction (%)	70 (66, 73)	48 (42, 55)*^†^	22 (17, 32)*	< 0.001
CMR				
LV-EDVI (mL/m^2^)	75.62 (65.7, 84.5)	80.62 (71.8, 88.0)^†^	138.69 (111.2, 162.3)*	< 0.001
LA-GLS (%)	18.8 (13.5, 23.9)	20.7 (16.4, 27.2)^†^	8.4 (4.8, 12.7)*	< 0.001
LAVImax (mL/m^2^)	64.0 (50.8, 81.4)	47.9 (39.9, 61.2)*^†^	66.0 (49.6, 81.8)	< 0.001
LAVImin (mL/m^2^)	34.9 (23.5, 48.4)	23.47 (17.58, 30.24)*^†^	49.35 (31.59, 66.94)*	< 0.001
LA preA volume index (mL/m^2^)	49.17 (33.91, 60.22)	36.4 (28.9, 44.3)*^†^	52.4 (37.0, 68.5)	< 0.001
LA total emptying fraction (%)	44.7 (35.1, 52.1)	51.2 (44.7, 59.1)*^†^	25.9 (20.2, 38.0)*	< 0.001
LA reservoir fraction (%)	80.7 (54.0, 108.7)	105.1 (80.8, 144.7)*^†^	35.0 (25.3, 61.3)*	< 0.001
LA conduit fraction (%)	27.0 (17.4, 36.9)	35.7 (21.6, 45.1)*^†^	16.4 (9.4, 27.2)*	< 0.001
LA active emptying fraction (%)	30.2 (23.8, 35.3)	35.3 (29.7, 41.8)*^†^	17.9 (9.0, 31.6)*	< 0.001
E/e′/LA-GLS (%)	0.7 (0.5, 1.3)	0.5 (0.4, 0.8)*^†^	1.9 (1.1, 4.3)*	< 0.001
ECVavg (%)	31.9 (28.8, 35.3)	37.2 (33.9, 42.2)*^†^	33.6 (30.2, 35.9)	< 0.001

^1^At the time of the echocardiography or CMR; ^2^NT-proBNPs were done in 29 HCM and 40 DCM patients, BNP in 39 AMI and 52 DCM patients;^.^a′, late diastolic mitral annular velocity; ACEi, angiotensin-converting enzyme inhibitor; AMI, acute myocardial infarction; ARB, angiotensin receptor blocker; BNP, B-type natriuretic peptide; CAD, coronary artery disease; CMR, cardiovascular magnetic resonance; DCM, dilated cardiomyopathy; e′, early diastolic mitral annular velocity; ECV, extracellular volume fraction; EDVI, end-diastolic volume index; eGFR, estimated glomerular filtration rate; GLS, global longitudinal strain; HCM, hypertrophic cardiomyopathy; LA, left atrial; LAVImax, maximal LA volume index; LAVImin, minimal LA volume index; LGE, late gadolinium enhancement; LV, left ventricular; NT-proBNP, N-terminal pro-BNP; NYHA, New-York Heart Association; PASP, pulmonary arterial systolic pressure; s′, systolic mitral annular velocity

*p < 0.0167 compared to HCM, ^†^*p* < 0.0167 compared to DCM

### Differential contribution of LA function and LV fibrosis to PASP

**Fig. 2:**
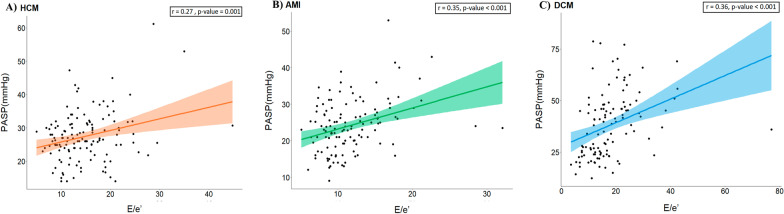
Subgroup clustering between E/e′ and pulmonary arterial systolic pressure in hypertrophic cardiomyopathy (**A**), reperfused acute myocardial infarction (**B**), and dilated cardiomyopathy (**C**). The correlations were modest in all groups. HCM, hypertrophic cardiomyopathy; PASP, pulmonary arterial systolic pressure; E/e′, ratio of early diastolic mitral inflow to annular velocity; AMI, acute myocardial infarction; DCM, dilated cardiomyopathy

A stepwise backward linear regression analysis showed that E/e′ was significantly but weakly correlated with PASP in all groups (Fig. 2); moreover, their relationship was significantly attenuated after adjusting for the LA volume and LA function in HCM, but remained significant in AMI. The LA stiffness index, E/e′/LA-GLS, in HCM [coefficient 1.1, 95% confidence interval (CI) 0.3–2.0, *p* = 0.01] and DCM (coefficient 0.6, 95% CI 0.06–1.2, *p* = 0.03) was significantly related to the PASP independent of LAVImax. In the DCM group, the average ECV was significantly correlated with the PASP (coefficient 1.1, 95% CI 0.5–1.6, *p* < 0.001) independent of the LAVImax and E/e′ (Table 2). The AMI group did not show any relationships, except for LV ejection fraction and E/e′ (Additional file 1: table S3). But, the relationship between PASP and E/e′ and LA-stiffness index was better in patients with LAD-territory AMI (Additional file 1: table S4).

**Table 2:** Differential contribution of the left atrial volume, function, and left ventricular fibrosis on the pulmonary arterial systolic pressure in hypertrophic cardiomyopathy and dilated cardiomyopathy

	HCM				DCM			
	Univariable analysis		Multivariable analysis		Univariable analysis		Multivariable analysis	
	B (95% CI)	*p* value	B (95% CI)	*p* value	B (95% CI)	*p* value	B (95% CI)	*p* value
Echocardiography								
LV-EF (%)	− 0.2 (− 0.4, − 0.04)	0.02	− 0.1 (− 0.3, 0.03)	0.09	− 0.3 (− 0.6, − 0.1)	0.005	− 0.2 (− 0.5, 0.02)	0.08
e′ (cm/s)	− 30.4 (− 105.6, 44.8)	0.43			− 114.3 (− 240.2, 11.6)	0.08		
s′ (cm/s)	− 83.7 (− 155.7, − 11.7)	0.02			− 157.2 (− 348.9, 34.4)	0.11		
E/e′	0.4 (0.1, 0.6)	0.001	0.1 (− 0.1,0.3)	0.30	0.6 (0.3, 0.8)	< 0.001	0.07 (− 0.2, 0.4)	0.66
CMR								
LA-GLS (%)	− 0.2 (− 0.4, − 0.1)	< 0.001	− 0.02 (− 0.3, 0.3)	0.87	− 0.7 (− 1.0, − 0.4)	< 0.001	− 0.2 (− 0.6, 0.3)	0.49
LAVImax (mL/m^2^)	0.1 (0.1, 0.2)	< 0.001	0.12 (0.07, 0.2)	< 0.001	0.2 (0.1, 0.3)	< 0.001	0.1 (0.03, 0.3)	0.02
LAVImin (mL/m^2^)	0.1 (0.1, 0.2)	< 0.001			0.2 (0.1, 0.3)	< 0.001		
LA total emptying fraction (%)	− 0.2 (− 0.3, − 0.1)	< 0.001			− 0.4 (− 0.6, − 0.2)	< 0.001		
LA reservoir fraction (%)	− 0.05 (− 0.08, − 0.03)	< 0.001	− 0.01 (− 0.04, 0.02)	0.43	− 0.1 (− 0.2, − 0.06)	< 0.001		
LV-ECVavg (%)	0.3 (0.04, 0.6)	0.03	− 0.04 (− 0.3, 0.3)	0.80	1.3 (0.7, 1,9)	< 0.001	1.1 (0.5, 1.6)	< 0.001
E/e′/LA-GLS (%)	1.8 (1.0, 2.7)	< 0.001	0.09 (0.06, 1.7)	0.04	1.0 (0.5, 1.6)	< 0.001	0.6 (0.06, 1.2)	0.03

See abbreviations in Table 1

### Characteristics of discrepantly higher or lower PASP group compared to E/e′

**Fig. 3:**
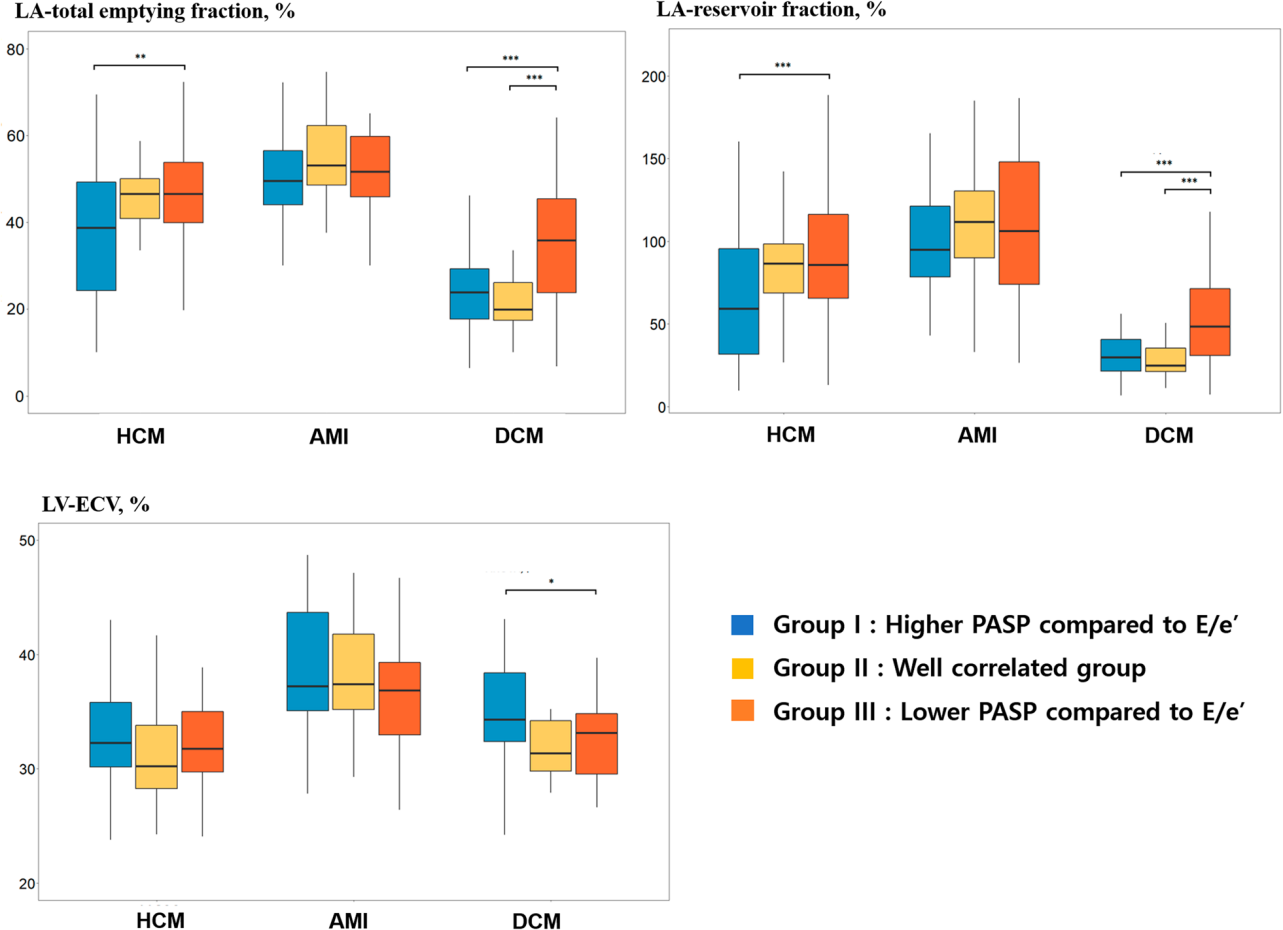
Comparisons of the left atrial functional indexes between subgroups according to E/e′ and pulmonary arterial systolic pressure. **A** LA total emptying fraction, %, **B** LA reservoir fraction, %, and **C** LV-ECV avg, %. Higher PASP compared to E/e′ (group I), patients on the regression line between PASP and E/e′ within 95% CI (group II), and lower PASP compared to E/e′ (group III). LA, left atrial; LV-ECVavg, average extracellular volume fraction of left ventricle; PASP, pulmonary arterial systolic pressure; *p < 0.05; **p < 0.01; ***p < 0.001 in ANOVA

When the patients were subdivided into three groups according to the linear regression between PASP and E/e′ in each disease entity, group I was defined as the patients with higher PASP compared to E/e′, group II as the patients on the regression line between PASP and E/e′ (within 95% CI), and group III as having lower PASP compared to E/e′. The patients with discrepantly high PASP (group I) had a higher LA preA volume index than those in group III in HCM. Group I also showed a lower total emptying fraction and reservoir fraction compared to group III (*p* < 0.05), without significant differences from group II. In reperfused AMI, there were no significant differences between the groups (Additional file 1: table S5). In DCM, the LA GLS, total emptying fraction, reservoir fraction, conduit fraction, and the active emptying fraction were the highest in group III (*p* < 0.05). The LA stiffness index was lowest in Group III. ECV was the highest in group I and showed significant differences between the subgroups (*p* < 0.05) (Table 3 and Fig. 3).

**Table 3:** Determinants of discrepantly higher or lower pulmonary arterial systolic pressure compared to E/e′

	Variables	Higher PASP compared to E/e′ (Group 1, n = 50)	On the regression line between PASP and E/e′ (Group 2, n = 28)	Lower PASP compared to E/e′ (Group 3, n = 55)	Overall *p* value
HCM	LA-GLS (%)	17.5 (10.5, 22.4)	18.7 (14.9, 23.0)	20.8 (15.1, 27.1)	0.063
	LAVImax (mL/m^2^)	80.9 (65.3, 98.2)	58.3 (45.3, 68.9)*	56.2 (46.7, 74.6)*	< 0.001
	LAVImin (mL/m^2^)	47.7 (33.2, 75.3)	30.4 (25.6, 38.8)*	30.0 (21.4, 39.5)*	< 0.001
	LA preA volume index (mL/m^2^)	59.1 (39.6, 74.2)	46.1 (33.4, 55.8)	43.2 (33.4, 55.9)*	0.011
	LA total emptying fraction (%)	37.2 ± 15.8	45.9 ± 8.8	45.9 ± 13.2*	0.002
	LA reservoir fraction (%)	61.3 (31.7, 96.6)	87.2 (69.4, 100.0)	88.5 (67.4, 119.5)*	0.014
	LA conduit fraction (%)	26.2 (18.2, 40.5)	26.0 (18.8, 33.0)	27.2 (17.2, 36.9)	0.855
	LA active emptying fraction (%)	27.7 ± 12.2	31.5 ± 8.9	31.8 ± 13.1	0.242
	E/e′/LA-GLS (%)	0.9 (0.5, 1.5)	0.6 (0.5, 0.9)	0.7 (0.6, 1.1)	0.219
	ECVavg (%)	33.1 ± 5.1	31.3 ± 4.8	31.9 ± 4.1	0.261
	LVMI (by TTE) (g/m^2^)	128.6 (108.8, 152.0)	105.01 (94.6, 120.0)*	121.6 (104.3, 135.4)	0.006
	LVEF (by TTE) (%)	69 (65, 72)	70 (65, 73)	70 (66, 73)	0.637

	Variables	(Group 1, n = 45)	(Group 2, n = 16)	(Group 3, n = 53)	
DCM	LA-GLS (%)	7.5 (3.5, 11.8)	6.7 (3.4, 8.0)^†^	11.5 (6.9, 23.1)*	0.001
	LAVImax (mL/m^2^)	76.1 (60.8, 108.3)	70.0 (63.7, 81.4)^†^	52.3 (41.6, 74.5)*	< 0.001
	LAVImin (mL/m^2^)	60.3 (44.3, 86.6)	53.7 (46.6, 68.9)^†^	34.8 (20.8, 51.5)*	< 0.001
	LA preA volume index (mL/m^2^)	63.6 (50.5, 84.9)	61.1 (51.7, 70.0)^†^	41.3 (29.9, 57.8)*	< 0.001
	LA total emptying fraction (%)	24.8 (17.7, 30.2)	20.0 (17.4, 26.1)^†^	36.1 (23.9, 50.0)*	< 0.001
	LA reservoir fraction (%)	33.0 (21.5, 43.3)	24.9 (21.1, 35.4)^†^	56.5 (31.3, 100.0)*	< 0.001
	LA conduit fraction (%)	15.5 (4.6, 21.5)	11.0 (9.4, 15.0)	19.7 (12.0, 33.7)*	0.006
	LA active emptying fraction (%)	12.1 (8.1, 20.4)	9.6 (8.3, 18.9)^†^	26.4 (16.0, 35.7)*	0.002
	E/e′/LA-GLS (%)	2.7 (1.3, 5.4)	4.1 (2.8, 6.0)^†^	1.3 (0.5, 2.4)*	0.011
	ECVavg (%)	35.4 ± 5.4	31.6 ± 2.5	32.5 ± 3.8*	0.005
	LVMI (by TTE) (g/m^2^)	134.3 ± 30.3	127.4 ± 31.5	125.6 ± 42.3	0.491
	LVEF (by TTE) (%)	20 (15, 26)	18 (15, 23)^†^	29 (20, 40)*	< 0.001

See abbreviations in Table 1; *p < 0.0167 compared to group I, ^†^*p* < 0.0167 compared to group III

## Discussion

To the best of our knowledge, this is the first study to evaluate the differential contribution of the LA function to PASP by CMR in HCM, DCM and reperfused AMI. This study elucidated several findings: E/e′ was significantly correlated with the PASP in all groups; however, their relationship was significantly attenuated after adjusting for the LAVI and LA strain, except in patients with reperfused AMI. This suggests that LAVI or LA function significantly contributes to the development of pulmonary arterial pressure elevation in the HCM and DCM groups.

### Differential contribution of LA function to PASP

LA stiffness increases with atrial remodeling and reflects a deteriorated atrial function. The LA volume and LA filling pressure are known to be independent predictors of LA stiffness. The LA stiffness index was calculated as the ratio of E/e′ to LA GLS, as demonstrated previously. It was noted to be the most accurate index for differentiating the patients with HFpEF from asymptomatic diastolic dysfunction [7]. Our study also supports that, in HCM, a prototype of HFpEF, the LA stiffness index was independently related to PASP. In addition, the LAVImax was significantly related to PASP, independent of E/e′ and LA stiffness. The LA volume plays a role in the morphophysiological expression of LV diastolic dysfunction [13]. Several studies have demonstrated that an increased LA size in patients with HCM is associated with impaired LA function [18]. Thus, our study further validated previous studies by detecting the correlation of LA stiffness index and LAVI with PASP in HCM patients. When the patients were subdivided according to the linear regression between PASP and E/e′, the patients in the discrepantly high PASP group (lower E/e′ but higher PASP) had lower total emptying fraction and reservoir fraction of LA in HCM in this study. In patients with HCM, the LA compliance is thought to be decreased due to an increased wall stiffness that results in LA dysfunction, as well as the significant contribution of LA function to PASP, and the presence of independent atrial myopathy in HCM. In AMI, the LA active emptying fraction was significantly lower in the higher PASP group than in group II. In prior studies, it was already known that the LA reservoir function is closely correlated with LV filling pressures [19]. The LA reservoir function and LA fractional changes were all mainly associated with LV diastolic and systolic dysfunction in AMI [20]. In our study, the LA reservoir fraction showed a tendency to decrease in the higher PASP group compared to group II in HCM and AMI. The LA physiologic phases are interrelated, and the occurrence of low reservoir function may be compensated by an increased active booster pump function [20]. Therefore, it is possible to hypothesize that if the active booster function of LA does not sufficiently compensate for impaired passive function, it could lead to an impaired reservoir function and LV filling dysfunction with higher PASP relative to E/e′ in reperfused AMI.

### Direct measurement of LV fibrosis as a component of diastolic function

In DCM, LAVImax, LA stiffness index, and the ECV of left ventricle were significantly related to PASP independent of E/e′. Our results also further confirm that E/e′ is poorly correlated to the pulmonary capillary wedge pressure in DCM; therefore, it suggests that the direct measurement of LV fibrosis amount would provide incremental information of LV diastolic function. In HFrEF, exposure to high LV filling pressure causes an increase in the LA volume. An increased LA volume often integrates the effects of a decline in both the systolic and diastolic function of the LV and also affects the right side of the heart, causing high pulmonary artery and venous pressures [21]. Furthermore, the degree of ECV by post-contrast T1 values was closely related to LV remodeling and diastolic function in DCM in a previous study [9]. According to our study results, we observed that diffuse LV fibrosis limits LV filling by impaired active LV relaxation and increased LV passive stiffness incorporation into LA remodeling. Thus, it might elevate the PASP. However, ECV in DCM has been shown to be in a similar range to HCM; thus, a question arises as to why ECV was related to PASP only in DCM, but not in HCM. The pathophysiological correlates that are responsible for similar ECV values and their effects on PASP in DCM and HCM require further study.

### Clinical and therapeutic perspectives

In our study, LA stiffness index by incorporating E/e′ and LA-GLS was found to have a significant effect on PASP both in HCM (prototype of HFpEF) and DCM (prototype of HFrEF) independently. However, the volumetric LA function parameters (LA emptying and reservoir fraction) did not have a independent effect on PASP in all of 3 groups. Therefore, LA stiffness index, which potentially incorporates pressure–volume relationship, might explain the impaired LA function better than the conventional volumetric method and the LA strain alone [4, 7, 8], and it is thought that it could predict PASP well in HCM and DCM, which had potential atrial myopathy. In HCM, LA stiffness could affect to the PASP, and LV fibrosis also could in DCM. Therefore, in HCM or DCM, it is necessary to specially control pressure or volume overload applied to the atrium. It is also known that AF is an important factor causing the increase in atrial stiffness. In this study, higher incidence of AF was observed in HCM and DCM compared to AMI group. Therefore, sinus conversion from AF could be very helpful to prevent further progression of LA myocardial dysfunction. In DCM, anti-fibrotic therapy using angiotensin converting enzyme inhibitor (ACEi), angiotensin II receptor/neprilysin inhibitor (ARNI), sodium glucose co-transporter 2 inhibitor (SGLT2i) and mineralocorticoid receptor antagonist (MRA) could be also helpful to prevent progression of LV diastolic dysfunction. It supports that longer term anti-fibrotic medications such as ARNI, ACEi, MRA and SGLT2i had favorable prognostic effects in DCM, a main cause of HFrEF [22]. Regarding HCM, it suggests a potential role of mevacamten which have anti-fibrotic effects [23] for the prevention of LA dysfunction. But, it warrants further research.

## Conclusion

Contrary to that in AMI, the LA stiffness representative of the LA function in both HCM and DCM contributes to the PASP independent of E/e′ and the LA size. Furthermore, LV fibrosis has an added effect on the LA function in patients with DCM. These results suggest the presence of atrial myopathy and the usefulness of LA functional measurements in non-ischemic cardiomyopathies and ECV measurement in DCM for the comprehensive evaluation of LV diastolic function.

### Limitations

Our study had several limitations. This was a single-center study with a relatively modest sample size. Thus, a subgroup analysis for each of the three groups may have a relatively weak statistical power. Furthermore, because of its invasiveness, cardiac catheterization was not performed; thus, the cardiac physiological parameters, including the LA pressure or pulmonary capillary wedge pressure were not measured. The pre-A volume could not be measured in some patients, especially in DCM due to the lower temporal resolution in CMR and reduced LA function; in these cases, echocardiography would be better for detecting subtle phasic changes in the pre-A period. However, CMR has better spatial resolution; therefore, mutual compensation and future technical developments are needed.

## Supplementary Information

**Additional file 1. Table S1**. Comparison of clinical, echocardiographic and CMR findings between left anterior descending coronary artery (LAD) and non-LAD territory acute myocardial infarction.** Table S2**. Relationship between average extracellular volume fraction of left ventricle and left atrial anatomic and functional parameters.**Table S3**. Differential contribution of the left atrial volume, function, and left ventricular fibrosis on the pulmonary arterial systolic pressure in reperfused acute myocardial infarction.** Table S4**. Relationship between pulmonary arterial systolic pressure and diastolic functional parameters according to coronary artery territory in reperfused acute myocardial infarction.** Table S5**. Determinants of discrepantly higher or lower pulmonary arterial systolic pressure compared to E/e’ in acute myocardial infarction.

## Data Availability

Datasets are presented in the main manuscript and additional supporting files were deposited google drive and freely available. https://drive.google.com/file/d/1GgWrWMs8dz_oc38TxVZM9gnZbfTc0zGT/view?usp=share_link.

## Abbreviations

LA: Left atrial
LV: Left ventricular
PASP: Pulmonary arterial systolic pressure
HCM: Hypertrophic cardiomyopathy
DCM: Dilated cardiomyopathy
AMI: Acute myocardial infarction
CMR: Cardiovascular magnetic resonance
GLS: Global longitudinal strain
ECV: Extracellular volume fraction
E/e′: Ratio of an early mitral inflow to mitral annular velocity
PCWP: Pulmonary capillary wedge pressure
TRV: Tricuspid regurgitant velocity
HF: Heart failure
HFpEF: Heart failure with preserved ejection fraction
HFrEF: Heart failure with reduced ejection fraction
AF: Atrial fibrillation
