# Supplementary material for: Effects of left atrial function on pulmonary arterial pressure in acute myocardial infarction, hypertrophic and dilated cardiomyopathy
Source: BMC Cardiovasc Disord. 2022 Nov 26;22:507. doi: 10.1186/s12872-022-02952-8 (PMC9701432; doi:10.1186/s12872-022-02952-8)
Supplement: Supplementary file 1 — Additional file 1. Table S1. Comparison of clinical, echocardiographic and CMR findings between left anterior descending coronary artery (LAD) and non-LAD territory acute myocardial infarction. Table S2. Relationship between average extracellular volume fraction of left ventricle and left atrial anatomic and functional parameters.Table S3. Differential contribution of the left atrial volume, function, and left ventricular fibrosis on the pulmonary arterial systolic pressure in reperfused acute myocardial infarction. Table S4. Relationship between pulmonary arterial systolic pressure and diastolic functional parameters according to coronary artery territory in reperfused acute myocardial infarction. Table S5. Determinants of discrepantly higher or lower pulmonary arterial systolic pressure compared to E/e’ in acute myocardial infarction. [file 12872_2022_2952_MOESM1_ESM.docx]

**Supplemental table 1**. Comparison of clinical, echocardiographic and CMR findings between left anterior descending coronary artery (LAD) and non-LAD territory acute myocardial infarction.

| Variables | LAD group  (n=82) | non-LAD group  (n=41) | P |
| --- | --- | --- | --- |
| **Age**, years | 55.7 ± 11.8 | 57.0 ± 12.9 | 0.411 |
| **Male**, n (%) | 76 (93) | 35 (85) | 0.197 |
| **Body surface area**, m^2^ | 1.85 ± 0.19 | 1.80 ± 0.17 | 0.166 |
| **Systolic BP**, mmHg | 114 (104,138) | 106 (100, 129) | 0.399 |
| **Diastolic BP**, mmHg | 73 (65, 80) | 69 (62, 77) | 0.075 |
| **Diabetes**, n (%) | 15 (18) | 12 (29) | 0.165 |
| **Hypertension**, n (%) | 40 (49) | 18 (44) | 0.609 |
| **ACEi/ARB**, n (%) | 68 (83) | 33 (81) | 0.739 |
| **Beta-blocker**, n (%) | 72 (88) | 32 (78) | 0.158 |
| **Diuretics**, n (%) | 4 (5) | 6 (15) | 0.062 |
| **Atrial fibrillation**, n (%) | 1 (1) | 2 (5) | 0.215 |
| **Echocardiography** |  |  |  |
| **e’**, cm/s | 7 (5,9) | 6 (5,7) | 0.244 |
| **a’,** cm/s | 9 (7,10) | 9 (7,10) | 0.352 |
| **s’,** cm/s | 7 (6,9) | 7 (6,8) | 0.092 |
| **E/e’** | 10.9 (9.0, 13.2) | 10.4 (8.6, 15.1) | 0.727 |
| **PASP**, mmHg | 24.3 (21.0, 28.7) | 23.8 (19.4, 28.1) | 0.229 |
| **LAVI,** ml/m2 | 21.7 (17.3, 26.6) | 23.9 (17.5, 28.9) | 0.009 |
| **LVMI,** g/m2 | 93.5 (77.9, 107.3) | 92.9 (76.0, 105.3) | 0.760 |
| **LV ejection fraction**, % | 44 (39, 50) | 55 (51, 58) | <0.001 |
| **CMR** |  |  |  |
| **LA-GLS,** % | 20.7 (15.4, 69.9) | 20.6 (17.7, 28.6) | 0.342 |
| **LAVImax**, mL/m^2^ | 44.0 (38.8, 58.2) | 51.9 (46.0, 66.7) | 0.003 |
| **LAVImin**, mL/m^2^ | 23.5 (17.2, 28.9) | 24.1 (17.7, 35.2) | 0.161 |
| **LA total emptying fraction**, % | 50.9 (44.0, 58.1) | 51.3 (44.7, 61.1) | 0.652 |
| **LA reservoir fraction**, % | 103.6 (78.5, 138.4) | 105.3 (80.8, 157.3) | 0.652 |
| **LA conduit fraction**, % | 33.3 (21.3, 43.6) | 38.4 (25.3, 50.4) | 0.212 |
| **LA active emptying fraction**, % | 34.9 (28.8, 41.8) | 37.2 (31.2, 42.8) | 0.362 |
| **E/e’/LA-GLS**, % | 0.55 (0.38, 0.80) | 0.44 (0.33, 0.69) | 0.311 |
| **ECVavg**, % | 37.8 (34.4, 43.4) | 35.9 (31.1, 37.9) | 0.038 |

**Supplemental table 2.** Relationship between average extracellular volume fraction of left ventricle and left atrial anatomic and functional parameters.

|  | **HCM** | | **AMI** | | **DCM** | |
| --- | --- | --- | --- | --- | --- | --- |
|  | **r** | **p** | **r** | **p** | **r** | **p** |
| **LA maximal volume** | 0.346 | <0.001 | -0.053 | 0.625 | 0.305 | 0.003 |
| **LA minimal volume** | 0.402 | <0.001 | 0.086 | 0.431 | 0.271 | 0.009 |
| **LA maximal volume index** | 0.352 | <0.001 | 0.009 | 0.932 | 0.363 | <0.001 |
| **LA minimal volume index** | 0.399 | <0.001 | 0.126 | 0.244 | 0.312 | 0.002 |
| **LA global strain** | -0.325 | <0.001 | -0.245 | 0.022 | -0.073 | 0.489 |
| **LA total emptying fraction** | -0.362 | <0.001 | -0.249 | 0.020 | -0.087 | 0.410 |
| **LA reservoir fraction** | -0.290 | 0.001 | -0.238 | 0.026 | -0.070 | 0.510 |
| **LA conduit fraction** | -0,244 | 0.013 | -0.210 | 0.058 | -0.192 | 0.144 |
| **LA active emptying fraction** | -0.193 | 0.051 | -0.099 | 0.376 | -0,021 | 0.876 |
| **LA stiffness index** | -0.548 | <0.001 | 0.319 | 0.003 | 0.135 | 0.201 |

**Supplemental table 3.** Differential contribution of the left atrial volume, function, and left ventricular fibrosis on the pulmonary arterial systolic pressure in reperfused acute myocardial infarction.

See abbreviations in Table 1

|  | **AMI** | | | |
| --- | --- | --- | --- | --- |
|  | **Univariable analysis** | | **Multivariable analysis** | |
|  | **B (95% CI)** | **P-value** | **B (95% CI)** | **P-value** |
| **Echocardiography** |  |  |  |  |
| LV-EF, % | -0.2 (-0.3, -0.04) | 0.01 | -0.2 (-0.3, 0.01) | 0.06 |
| e’, cm/s | -22.2 (-83.0, 38.5) | 0.47 |  |  |
| s’, cm/s | -63.4 (-126.1, -0.8) | 0.05 |  |  |
| E/e’ | 0.6 (0.3, 0.9) | <0.001 | 0.5 (0.1, 1.3) | 0.02 |
| **CMR** |  |  |  |  |
| GLS_LA, % | -0.1 (-0.3, 0.0) | 0.05 | -0.04 (-0.3, 0.2) | 0.79 |
| LAVImax, mL/m^2^ | 0.04 (-0.04, 0.1) | 0.31 |  |  |
| LAVImin, mL/m^2^ | 0.1 (-0.02, 0.2) | 0.09 |  |  |
| LA total emptying fraction, % | -0.1 (-0.2, 0.01) | 0.08 |  |  |
| LA reservoir fraction, % | -0.02 (-0.05, 0.0) | 0.11 |  |  |
| LV-ECVavg, % | 0.3 (0.04, 0.5) | 0.02 | 0.06 (-0.2, 0.3) | 0.62 |
| E/e’/LA-GLS, % | 4.7 (2.0, 7.3) | 0.001 | -0.2(-9.4, 4.5) | 0.48 |

**Supplemental table 4.** Relationship between pulmonary arterial systolic pressure and diastolic functional parameters according to coronary artery territory in reperfused acute myocardial infarction.

|  | **LAD territory group** | | **Non-LAD territory group** | |
| --- | --- | --- | --- | --- |
|  | **r** | **p** | **r** | **p** |
| **E/e’** | 0.468 | <0.001 | 0.240 | 0.131 |
| **LA maximal volume index** | 0.183 | 0.100 | 0.037 | 0.817 |
| **LA minimal volume index** | 0.244 | 0.027 | 0.078 | 0.630 |
| **LA global GLS** | -0.157 | 0.160 | -0.229 | 0.149 |
| **LA total emptying fraction** | -0.191 | 0.085 | -0.093 | 0.563 |
| **LA reservoir fraction** | -0.188 | 0.091 | 0.063 | 0.706 |
| **LA active emptying fraction** | -0.261 | 0.021 | -0.041 | 0.807 |
| **LA stiffness index** | 0.386 | <0.001 | 0.228 | 0.152 |

LAD, left anterior descending coronary artery; LA, left atrial

**Supplemental table 5.** Determinants of discrepantly higher or lower pulmonary arterial systolic pressure compared to E/e’ in acute myocardial infarction

|  | Variables | Higher PASP compared to E/e’  (Group 1, n=43) | On the regression line between PASP and E/e’  (Group 2, n=35) | Lower PASP compared to E/e’  (Group 3, n=45) |  |
| --- | --- | --- | --- | --- | --- |
| AMI | **LA-GLS**, % | 18.7 (15.2, 26.7) | 20.9 (16.4, 27.1) | 21.2 (17.9, 27.2) | 0.442 |
|  | **LAVImax**, mL/m^2^ | 47.6 (39.7, 60.5) | 48.8 (38.8, 60.1) | 48.3 (41.1, 61.8) | 0.653 |
|  | **LAVImin**, mL/m^2^ | 23.4 (18.2, 29.4) | 24.4 (15.3, 29.2) | 24.0 (18.6, 30.9) | 0.451 |
|  | **LA preA, volume index,** mL/m^2^ | 36.1 (28.9, 45.1) | 36.7 (26.7, 43.5) | 36.7 (30.3, 47.5) | 0.668 |
|  | **LA total emptying fraction**, % | 50.2 ± 9.8 | 54.0 ± 11.0 | 50.1 ± 10.9 | 0.199 |
|  | **LA reservoir fraction**, % | 98.4 (78.8, 130.0) | 113.6 (94.6, 165.4) | 106.1 (73.8, 148.0) | 0.220 |
|  | **LA conduit fraction**, % | 34.8 (26.4, 44.6) | 35.7 (27.4, 48.2) | 36.2 (18.3, 43.4) | 0.661 |
|  | **LA active emptying fraction**, % | 32.7 ± 8.8 | 38.6 ± 10.9* | 35.7 ± 8.5 | 0.030 |
|  | **E/e’/LA-GLS, %** | 0.5 (0.4, 1.0) | 37.6 (35.3, 43.0) | 36.9 (33.1, 39.5) | 0.413 |
|  | **ECVavg, %** | 37.2 (35.1, 45.2) | 37.6 (35.3, 43.0) | 36.9 (33.1, 39.5) | 0.413 |
|  | **LVMI (by TTE)**, g/m^2^ | 91.1 (77.3, 103.6) | 96.6 (83.8, 107.4) | 94.5 (75.1, 107.6) | 0.718 |
|  | **LVEF (by TTE)**, % | 46.9 ± 10.1 | 49.9 ± 9.8 | 50.1 ± 10.2 | 0.262 |

See abbreviations in Table 1; *p<0.0167 compared to group I, †p<0.0167 compared to group III
